# Red Blood Cells and Hemoglobin in Human Atherosclerosis and Related Arterial Diseases

**DOI:** 10.3390/ijms21186756

**Published:** 2020-09-15

**Authors:** Jean-Baptiste Michel, José Luis Martin-Ventura

**Affiliations:** 1 UMR 1148, Inserm & Paris University, X. Bichat University Hospital, 75018 Paris, France; 2 IIS-Fundation Jimenez-Diaz, Autonoma University of Madrid and CIBERCV, 28040 Madrid, Spain

**Keywords:** evolution, iron, oxidation, frictional forces, atheroma, intraplaque hemorrhages, clotting, aneurysm, calcification, infarction, stroke, senescence, inhibitors, anti-oxidant

## Abstract

As the main particulate component of the circulating blood, RBCs play major roles in physiological hemodynamics and impact all arterial wall pathologies. RBCs are the main determinant of blood viscosity, defining the frictional forces exerted by the blood on the arterial wall. This function is used in phylogeny and ontogeny of the cardiovascular (CV) system, allowing the acquisition of vasomotricity adapted to local metabolic demands, and systemic arterial pressure after birth. In pathology, RBCs collide with the arterial wall, inducing both local retention of their membranous lipids and local hemolysis, releasing heme-Fe^++^ with a high toxicity for arterial cells: endothelial and smooth muscle cells (SMCs) cardiomyocytes, neurons, etc. Specifically, overloading of cells by Fe^++^ promotes cell death. This local hemolysis is an event associated with early and advanced stages of human atherosclerosis. Similarly, the permanent renewal of mural RBC clotting is the major support of oxidation in abdominal aortic aneurysm. In parallel, calcifications promote intramural hemorrhages, and hemorrhages promote an osteoblastic phenotypic shift of arterial wall cells. Different plasma or tissue systems are able, at least in part, to limit this injury by acting at the different levels of this system.

## 1. Introduction

On the one hand, red blood cells (RBCs) or erythrocytes are the most abundant (4.5 10^6^/mm^3^) circulating cells (90%). Platelets are the second most abundant (300,000/mm^3^), and polymorpho-nuclear leucocytes (PMNs, <8000/mm^3^) are the third. On the other hand, the arterial structures are permanently submitted to mechanical, biochemical and cellular stress, always initiated by hemodynamics, responsible for repeated wall injuries [1]. The purpose of the present synthesis is to describe the physiology and pathologies of the human arterial wall, directly involving RBCs and their derived membranous and cytosolic products. Therefore, we review observational data in humans, focusing on the presence of RBCs, hemoglobin and redox-active iron, and try to explain how RBCs, and particularly intratissue hemolysis and Fe^++^-derived oxidative stress, are cytotoxic and directly participate in these human pathologies. Finally, RBCs and their derivatives are omnipresent in all cardiovascular (CV) and neurovascular (NV) pathologies.

## 2. How Phylogeny and Ontogeny Define Red Blood Cells (RBCs) in the Human Circulation

The circulation in invertebrates is composed of a myoepithelial tube (heart) generating motion of the hemolymph in an archaic circulatory system, directly open to the extracellular fluid. Hemolymph is composed of a fluid phase (plasma and interstitial fluid) and a cellular phase: hemocytes. Hemocytes are nucleated cells, mainly involved in innate immunity [2] and coagulation in invertebrates. Oxygen transport is carried out more by plasma proteins such as hemocyanin (a blue copper-protein) than by hemocytes, which contain only a small quantity of hemoglobin (a red ferrous iron-protein). For instance, the ability of the horseshoe crab amoebocyte to clot (Limulus test), used for the sensitive detection of liposaccharides of bacterial origin [3], provides evidence of the role of amoebocytes in innate immunity and its mediation by clotting in invertebrates.

In fish (lower vertebrates), erythrocytes are nucleated, containing cytosolic organelles, carrying hemoglobin (Hb) and distributing oxygen to the body. However, they also actively participate in immunity, being involved in pathogen recognition, binding and clearance, particularly in antiviral defense [4], as an imprinting of the invertebrate stage.

In mammalian embryos, nucleated erythroblasts appear at three weeks and are produced by the yolk sac. During fetal life, the initial liver production shifts to bone-marrow, from five months until birth. After birth, the bone marrow remains the only site of hematopoiesis. Nucleated erythroblasts are derived from pluripotent hematopoietic progenitor cells [5], which are progressively loaded by synthesis of globin and iron recycling. Finally, the nucleus is expelled by an actin-dependent mechanism. Each erythrocyte is loaded with 30 pg of Hb. Besides RBC hemoglobin, other tissue goblins exist such as myoglobin, neuroglobin and cytoglobin, genetically derived from ancient hemoglobin. They predominantly serve for tissue oxygen storage, particularly in marine mammals. The mean life of erythrocytes is 120 days. In laminar rheology, RBCs flow in the center of the blood stream at a distance from the wall, whereas low-mass platelets flow in the periphery, close to the endothelium [6,7]. RBCs (8-μm diameter) are highly deformable, allowing them to penetrate very small channels, such as the 0.5-μm-wide slits in the reticulo-endothelial system of the spleen red pulp, and adapt their form to the circulating shear rate [8]. This property is linked, at least in part, to the enrichment of the RBC membrane in cholesterol with a high ratio of cholesterol to phospholipids. Non-laminar flow causes loss of this property and brings the RBCs close to the arterial wall.

With time, anucleate erythrocytes progressively expose phosphatidylserine (PS) on their external membrane, as a signal of senescence recognition. Fluorescent Annexin V binding in flow cytometry can be used to quantify this senescence [9]. Senescent RBC clearance [10] by phagocytes (spleen macrophages) is dependent on PS exposure and physiologically takes place in the red pulp of the spleen (for details, see Section: Clearance of senescent RBC physiology/pathology). In this context, transfusion experiments with fresh or conserved old blood provide an informative lesson about the relationship between RBC/Hb and the arterial tissue. There is evidence that blood submitted to a long storage before transfusion is not well supported in humans [11]. Experimental models of long versus short storage confirmed and extended these observational data. Baek and coworkers [12] performed an outstanding study comparing 2-day to 21- and 28-day RBC storage in guinea-pigs. The animals perfused with “senescent blood” presented a higher level of hemolysis with free hemoglobin within plasma, an acute hypertension mainly related to NO inhibition by free Hb, a decrease in RBC deformability, an increase in methemoglobin (Fe^+++^ Hb) and kidney and arterial injury (necrotic localized lesions). Finally, ferrous (Fe^++^) redox-active iron accumulates in liver and spleen, as well as in the aortic wall. A majority of these detrimental effects are prevented by the co-administration of haptoglobin (Hp). Sickle cell anemia and malaria attacks share some similarities with this pathophysiology. These data demonstrate that senescent RBCs are frail and more sensitive to hemolysis and that their detrimental cytotoxicity on the arterial wall is mainly mediated by hemolysis and the powerful oxidative capacity of heme-ferrous iron. For instance, the focal external application of FeCl_3_ is a usual model to induce experimental clotting in arteries. This model involves the inward cellular transport of FeCl_3_ towards the endothelium and is dependent on local hemolysis of circulating RBCs [13]. Formation of endothelial RBC aggregates, hemolysis and loss of endothelium precede platelet activation and thrombus formation in this model [14]. This model underlines the importance of the interaction between redox-active iron, de-endothelialization and clotting in the CV system. Therefore, it is necessary to detect redox-active Fe^++^ in tissue submitted to local hemolysis.

## 3. Frictional Forces Involve RBCs and Interact with Nitric Oxide (NO)

Blood is a viscous fluid moving in the circulation due to the kinetic energy generated by the heart’s pumping ability. Due to its high viscosity (51 ± 15 mPa), blood motion along the arterial tree generates frictional forces, which can transform kinetic energy (flow and shear stress) into potential energy (pressure), by the law of mechanical energy conservation in a closed homeothermic system (Bernoulli’s principle) and dissipation of both kinetic and potential mechanical energy upstream to the capillary capacitive compartment. The first experimental description of frictional forces in the dog mesenteric arteries was made by Stephen Hale (1677–1761, Statical Essays containing haemostaticks; London 1733). The relationship among pressure, flow velocity, viscosity and arterial radius is defined by the Hagen–Poiseuille law in which peripheral resistance to flow is directly proportional to viscosity (1/1) and inversely proportional (1/r^4^) to the fourth power of the radius [1]. Therefore, changes in the radius are the most important determinants of variations in frictional forces, responsible for blood pressure, in association with viscosity. Blood viscosity components are dependent on both circulating cell density and plasma protein concentration. Since RBCs represent 90% of the circulating cells, in physiological conditions, the hematocrit is classically considered as the main determinant of blood viscosity.

Viscosity-dependent shear stress is also the main physiological activator of endothelial NO synthase. NO promotes smooth muscle cell (SMC) relaxation by inhibition of vasomotor tone, inducing functional dilation of the artery (local regulation of vasomotricity). Free Hb or heme are potent local or systemic chelators of NO, enhancing SMC contraction and arterial vasomotor tone within tissue (vasospasm), or systemic hypertension [15]. Heme from free Hb scavenges NO and forms nitrosoHb, promoting the shift of Fe^++^ (ferrous) to Fe^+++^ (ferric), forming metHb, which cannot bind oxygen, and nitrite release as an intermediate metastable metabolite rapidly converted to nitrate as the definitive stable metabolite [16]. However, NO can also be stored and transported by binding to cysteine 93 of the globin β−chain (SNOHb). NitrosoHb predominates. Moreover, nitrite can be reconverted to NO [17] by mitochondrial nitrite reductase in relation to hypoxia [18]. These NO pathways in interaction with Hb are mainly involved in hypoxia-induced vasodilation [18], but not in physiological shear endothelium-dependent vasodilation in which the inhibitory role of free Hb and nitrate formation predominates. Moreover, cytoglobin (Cygb), an ancient hemoglobin isoform, plays also a major role in the modulation of NO activity [19]. Cygb is the predominant globin in arterial SMCs. It has a powerful ability to metabolize NO due to its dioxygenase activity [20]. When NO reacts with the oxygen bound to Cygb-heme, it produces nitrate and the oxidative shift from the Fe^++^ to the Ferric Fe^+++^ reduced state, bound to ferritin (storage and recycling). Cygb has the capacity to act both functionally, by limiting in part the SMC relaxation caused by endothelial NO, and structurally. Cygb suppression in mouse inhibits SMC intimal proliferation in vivo and promotes SMC apoptosis in vitro [21].

In parallel, free Hb is highly toxic for endothelial cells [22]. Important frictional forces, involving RBCs colliding with endothelial cells are also, with time, the main determinant of endothelial abrasion in conductance arteries [23]. Exposure of circulating blood to subendothelial glyco-aminoglycans [24,25] and collagen promotes platelet activation and aggregation and finally RBC clotting. Erosion is relatively specific of the coronary circulation (not observed in carotid arteries for instance) and predominates in epicardial conductance arteries, since shear stress is high in protodiastole, when the left ventricle relaxes, and flow abruptly invades the myocardium. These cyclic transitory high frictional forces occur around 3.10^9^ times during a lifespan of 80 years [1] and are responsible for endothelial abrasion. Erosion can cause localized de-endothelization and potentially acute coronary syndrome. This specific pathology predominates in women [26], potentially linked to the hormonal environment [27] and/or a high coronary flow rate during pregnancy and preeclampsia [28].

## 4. RBCs Colliding with the Arterial Wall

The relationship of atherosclerosis to arterial wall RBC accumulation, membrane cholesterol release forming cholesterol crystals [29], in situ hemolysis and powerful oxidative stress [30] are now well established. As described above, the evolution of the circulation from an “in series” closed system to an “in-parallel” system, with numerous changes in arterial wall geometry, has caused impingements of flow [1]. These changes in geometry induce loss in flow laminarity and an increased entropy (internal energy instability and dissipation) of the particulate part of the blood, promoting collision of blood cells with the wall and between themselves. The impact of colliding blood cells on the wall is mainly determined by the angulation of the bifurcation and luminal narrowing, but also depends on the hemorheology of circulating cells. The forces of collision impacting the wall are proportional to the mass (m) of the interacting RBCs and the velocity (v) at each point, corresponding to the mechanical quantity of movement (F = m.v). RBCs collide with the irregular geometry of the wall in participation or association with other biomechanical forces operating on the wall and drive intimal tears, micro-fissures and the formation of small mural hematomas. Collision of circulating cells among themselves is usually related to luminal dilation, associated with blood stagnation and vortices promoting endovascular clotting [31].

As proposed above, the phylogeny of circulating cells, involving hemocytes, innate immunity and clot formation, potentially imprints the behavior of these cells and the role of fibrinogen in mammals. In vivo, pure intra-tissue bleeding and the formation of mural hematoma without fibrin processing and coagulation does not exist. This is due to coagulation factor III (usually tissue factor, TF), a cell transmembrane protein, predominantly present on SMCs and on adventitial fibroblasts, in the arterial wall. TF initiates the extrinsic pathway of the coagulation [32]. This pathway leads to conversion of plasma prothrombin into active thrombin and finally to fibrin reticulation. These tissue and plasma cascades actively participate in physiological hemostasis, causing RBC clotting.

Conversely, thrombus formation without RBC entrapping within the fibrin network can occur in vivo (white clots including platelets, leukocytes and fibrin formation), but is unusual and a very large majority of thrombi are red (cruoric), due to the presence of RBCs within the fibrin network. Initial clotting is frequent in CV pathologies, due the platelet activation by the wall structural components (mainly collagen), including both plaque fissuration [33] and erosion [34]. The secondary fibrin reticulation entraps RBCs within the thrombi. Clotting can also be the consequence of blood stagnation and collision between circulating cells, related to dilation of the wall and hemodynamic vortices (aneurysm).

Therefore, the roles of RBCs in human atherosclerosis and related diseases are always intricately related with fibrin formation, fibrinolysis, platelet activation and fibrin entrapping of other circulating cells, mainly neutrophils [35]. Clotting of RBCs causes delayed hemolysis and release of membranes and Hb. Then, constituted clot evolves from red to brown and yellow, reflecting the transformation of hemoglobin, its oxidation and degradation. Until now, the relationship of RBCs to atherosclerosis was focused on tissue cholesterol enrichment by RBC membranes [29,36] in relation to intraplaque hemorrhages and plaque vulnerability [37]. In the present synthesis we focus on hemolysis occurring within tissues, associating extra and/or intracellular release of free hemoglobin and heme, which catalyze oxidative reactions by Fe^++^ as an electron donor. This pro-oxidant effect is not limited to the late stages (vulnerable plaque) [38] but also includes the initial stages of human atheroma and other associated acquired diseases.

In contrast, ferric iron (Fe^+++^), the ferritin-bound storage form of iron, is essentially neutral, without direct oxidative capacity. This contrast between heminic (ferrous) and ferric iron is exemplified by hemochromatosis in human. Hemochromatosis is a disease due to an increase in ferric iron intestinal absorption, leading to important iron storages in the liver, pancreas, spleen and myocardium [39,40]. Absorbed iron under the control of hepcidin is intracellularly transported by ferritin and extracellularly by transferrin, maintaining its redox-inactivity. Contrasting with hemolysis, hemochromatosis does not injure the arterial wall, and hemochromatosis is not an independent risk factor for atherosclerosis [41,42,43]. These observational data in human highlight that the behavior of Fe^++^ and Fe^+++^ is quite different in living tissues.

## 5. Presence of RBCs and Redox-Active Ferrous Iron in Different Human Arterial Pathologies

### 5.1. Methods for Ionized iron Detection in CV Tissues

Because RBCs contain Fe^++^-rich hemoglobin, fresh RBCs are able, in the presence of hydrogen peroxide (H_2_O_2_, Haber–Weiss and Fenton reactions), to produce free radicals and to oxidatively polymerize diaminobenzidine (DAB) into polybenzimidazole, forming a stable brown colored fiber, which precipitates within the tissue. This capacity disappears with ex-vivo time. In contrast, ferric iron (Fe^+++^) usually chelated by ferritin in vivo (redox inactive) is not directly able to polymerize DAB, but reacts with ferrocyanide, which precipitates as ferricyanide (Prussian blue, Perl’s reaction). The double reaction, first with ferricyanide and secondly, DAB polymerization in the presence of H_2_O_2_, is able to stain the redox-active Fe^++^, reflecting the oxidative imprinting of heme within the arterial tissue. It can also be revealed by ferricyanide in the presence of H_2_O_2_ (Turnbull reaction) [44]. The oxidative power of Fe^++^ is directly linked to its ability to promote electron transfer to superoxide anions or hydroxyl radicals (O_2_·^−^ and OH·+ Haber–Weiss and Fenton reactions). In these two reactions, redox-active Fe^++^ is the electron donor, catalyzing numerous oxidations of organic compounds such as lipids, proteins and DNA. Glycophorin A, a specific protein of the RBC, could also be used for detection or imprinting of cholesterol-rich RBC membranes within tissues. On histological sections, the co-localization of glycophorin A staining with cholesterol clefts (cholesterol crystals) suggests that these cholesterol clefts come from RBC membrane compaction within the vascular tissue.

In this tissue context, the formation of autofluorescent ceroids are markers of the ability of free heme to promote oxidation of lipids and proteins, forming an insoluble precipitate within the tissue. Since heme and hemoglobin spontaneously auto-fluoresce at 550 nm, ceroids are easily detected at this wavelength without staining. Ceroids can appear as punctiform cellular or extracellular autofluorescence, or as rings, reflecting a more advanced stage. For instance, ceroids can directly polymerize and precipitate DAB, providing evidence of heme-dependent oxidation. They can be also stained by oil red O on deparaffined sections, providing evidence of lipid entrapping in these insoluble aggregates.

### 5.2. Early Stages of Atheroma

**Figure 1:**
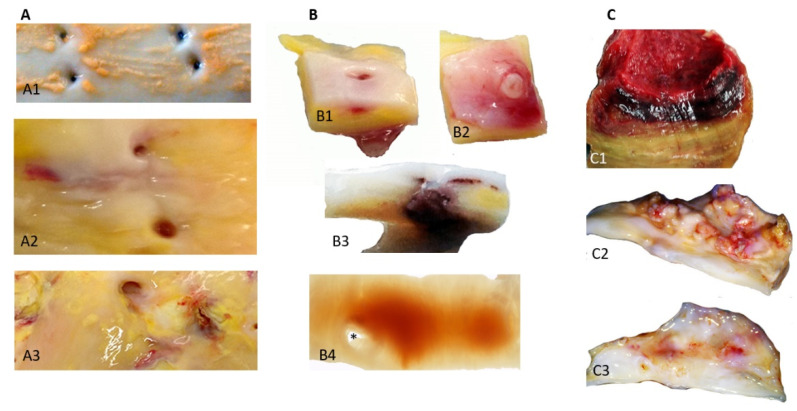
Macroscopic view of hemorrhages in human arterial lesions: (**A**) involvement of RBCs in the initial stages of human atherosclerotic lesions ((**A1**) aortic fatty streaks in human aorta and their relationship to intercostal ostia; (**A2**) fibro-atheroma with limited subintimal bleeding; (**A3**) more advanced lesions with numerous small hemorrhages and intimal breaches); (**B**) 0stial hematoma ((**B1**) ostial luminal view; (**B2**) outside collateral view; (**B3**) longitudinal trans-ostial section showing the local diffusion of the hematoma; and (**B4**) whole-mount immunostaining of Glycophorin A in peri-ostial clarified aortic tissue showing the hematoma diffusion from the ostia (*)); and (**C**) ILT in AAA and aortic valve; (**C1**) the multilayered intraluminal thrombus (ILT) of human AAA, where the most luminal layer (red) is extremely rich in RBCs and the subjacent layers are yellow/brown, demonstrating the release of heme and ferrous iron, and the metabolism of hemoglobin into bilirubin; (**C2**) aortic view of a calcified aortic valve, with peri-calcification hemorrhages (red), where the macro-crystallized calcifications protrude from the fibrosa; and (**C3**) view of the ventricularis of the same valve where the ventricularis surface is smoother than that of the fibrosa and the neo-angiogenesis network is visible (red)).

Classically, the first step of human atherosclerosis is the deposit of lipids (fatty streaks, FS) in the most luminal subendothelial layer, where circulating plasma apolipoproteins (apo), involving apo B, low density lipoproteins (LDL) and lipoprotein, Lp(a), directly interact with the hydrophilic GlycosAmino Glycans [45] synthesized by the intimal SMCs. In this lipid environment, SMCs are able to engulf LDL forming foam cells that accumulate lipid vesicles. The mass transport of lipoproteins from plasma to the wall is dependent on the outward hydraulic advective conductance driven by the pressure gradient between the circulating blood (80–120 mmHg) and adventitial interstitium (10–40 mmHg). This is why atherosclerosis is specific to the arterial part of the circulation, depending on the arterial hemodynamics, and is not observed in the venous system [46]. In the aorta, FS develop preferentially in the near proximity of intercostal or lumbar ostia, where the subendothelial lipid deposits lift up the endothelial layer creating irregularities. Early on, it was proposed that free heme has a detrimental effect on endothelial cells via its oxidant capacity [47,48] and catalyzes the peroxidase activity of neutrophils. This small enhancement of the endothelial layer is potentially enough to induce collision of RBCs with the wall (Figure 1A). In parallel, the first hematomas also appear in the angulation of these ostia (Figure 1B), providing evidence of the importance of specific local hemodynamics in the development of atherosclerosis by both increasing outward transport of plasma lipoproteins to the wall and promoting localized RBC collisions and hemolysis. These FS and early hematomas are progressively covered over by migrating SMCs that proliferate and synthesize extracellular matrix (ECM), producing the fibro-cellular cap. This healing process defines the second step in atheroma progression, plaques of fibro-atheroma (FA) with a lipid core and the cap. The lipid core is mainly acellular, due to the death of the SMCs. At this stage, whatever the source of lipids, lipoproteins or RBC membranes, the high density of tissue cholesterol forms crystals, liquid crystals which predominate in fatty streaks, progressing towards solid crystals which predominate in FA [49]. A similar process has been observed in coronary arteries.

**Figure 2:**
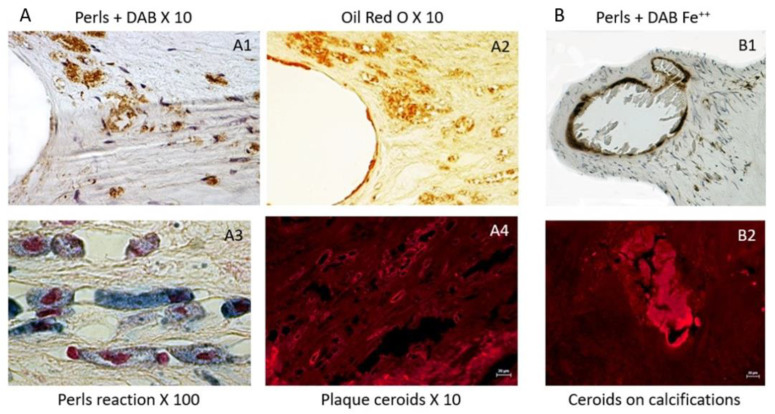
Histological views of tissue hemolysis in human aortic plaques and valves: (**A**) atherosclerosis ((**A1**) Perl’s + DAB staining of redox-active Fe^++^ in the shoulder of an atherosclerotic plaque; (**A2**) ORO lipid staining, colocalizing with Fe^++^ on a serial section; (**A3**) high magnification of foam SMCs by Prussian blue precipitation, showing the presence of Fe^+++^, potentially linked to ferritin (redox-inactive); and (**A4**) autofluorescent (550-nm wavelength, red) ceroid rings in an atherosclerotic plaque); and (**B**) calcified aortic valves ((**B1**) a crown of ferrous iron around calcification in an aortic valve, suggesting a compliant mismatch between the solid calcification and the soft elastic valve tissue; and (**B2**) red autofluorescence (550-nm wavelength) directly associated with aortic valve calcification).

**Figure 3:**
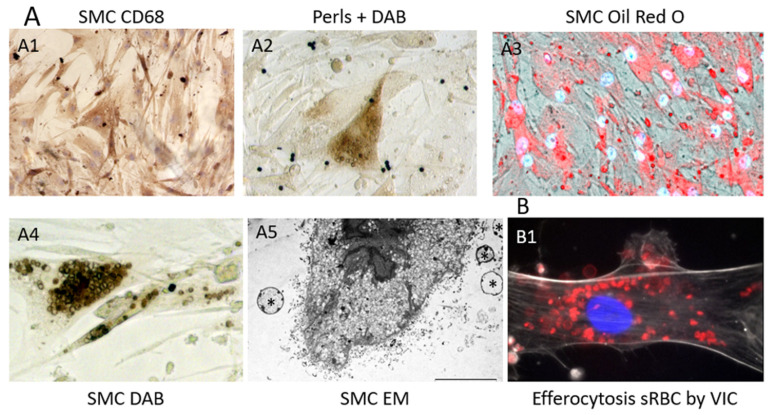
Phagocytosis of senescent RBCs by cultured human SMCs: (**A**) SMCs exposed to senescent sRBCs ((**A1**) acquisition of CD68, the phagolysosome functional marker of phagocytosis activity by cultured SMCs, usually used as a marker of macrophages, but not lineage-specific; (**A2**) acquisition of intracellular Fe^++^ staining; (**A3**) acquisition of lipid staining from sRBC membranes within SMCs; (**A4**) early phagocytosis of intact sRBCs, stained by DAB alone (pseudo-peroxidase activity); and (**A5**) electron microscopic view of efferocytosis of sRBCs by cultured human SMCs with the presence of extracellular RBC skeletons (*), containing hyperdense bodies, potentially iron and extracellular release of numerous micro-vesicles and exosomes by the sRBC-phagocytic SMCs); and (**B**) valvular Interstitial Cell (VIC) ((**B1**) efferocytosis of sRBCs (red) by human VIC).

The constitution of a biobank of healthy and early or late pathological human cardiovascular tissues, provides opportunities to compare advanced pathologies to early stages and healthy arterial walls and hearts, including coronary arteries (PFS 09-007, BRIF BB-0033-00029; BBMRI-EU/infrastructure BIOBANK). Using this original and rare human material resource, Delbosc and coworkers [9] observed the presence and demonstrated the role of RBCs and ferrous iron release in the initial stages of atheroma in human aorta. Using ferrocyanide, H_2_O_2_ + DAB polymerization (see above), they revealed the presence of redox-active Fe^++^ mainly present in foam cells, whereas the redox inactive, ferritin-stored, Fe^+++^ was detectable by the Perl’s reaction alone in these tissues and also in foam cells. The redox active Fe^++^ was associated with the presence of Hb, which colocalized with SMC myosin, suggesting that SMCs could endocytose hemoglobin or phagocytose RBCs. This process was associated with the presence of lipids (Oil Red O), autofluorescent (550 nm) red granules and small rings (ceroids) and glyphorin A (RBC membranes), providing evidence that RBCs penetrate early into the aortic intima and release hemoglobin and heme-iron (Figure 2). The quantity of iron present in the intimal tissue significantly increased with the progression from FS to FA. These foam SMCs were CD68 positive, showing activation of their phagolysosome. In this context, tissue redox-active iron and hemoglobin was increased in FS as compared to healthy aorta, but less than in FA, providing evidence that RBC-dependent release of redox-active iron is associated with the progression of the atheroma. As a consequence of this iron-dependent oxidative injury, the level of oxidized lipids and proteins were significantly increased in the intima of early stages of atheroma. Conversely, an increased tissue expression of natural resistance-associated macrophage protein (NRAMP), ferritin, ferroportin, hepcidin, heme-oxygenase (HO-1) and mannose receptor was associated with this hemolytic oxidative injury. This human tissue approach was completed by a cellular one in which human SMCs were cultured in the presence of fresh or senescent (PS-exposing) RBCs (Figure 3). Similar tissue observations were made in early stages of atherosclerosis in human coronary arteries. These data extend preceding data showing the presence of transition metal ions, mainly iron, in FS and their correlation to cholesterol accumulation [50]. In this context, redox-active iron could play a major role in the early oxidation of LDL [51]. These data are also important because the passage of a lipid into a necrotic core, involving cell death, is an important step in the evolution of atheroma towards the clinical expression of the disease. The cytotoxicity of free hemoglobin, releasing redox-active Fe^++^, promoting endoplasmic reticulum stress and oxidation of lipids, proteins and DNA, is now well established [52].

A specific iron-dependent, non-apoptotic process of cell death, named ferroptosis, has been described in cancer cells [53]. Ferroptosis involves the presence of redox-active iron, lipid peroxidation, mitochondrial fission and cell detachment, and it is largely dependent of non-enzymatic reactions [54]. This process was rescued by iron chelation (deferoxamine) and anti-oxidants [55]. This cytotoxic pathway could also be induced by ferric iron. It necessitates the entrance into the cell of the Fe^+++^/transferrin complex via the transferrin receptor and its reduction in Fe^++^ by endosomal ferric reductase at pH 5.2–5.6. It was recently shown that smoking-induced SMC toxicity in the CV system is mediated by ferroptosis [56]. Therefore, both SMCs and endothelial cells could be targets for ferroptosis [57]. To the best of our knowledge, the role of ferroptosis in the impact of hemoglobin-generated ferrous iron on the arterial wall remains to be explored.

### 5.3. Neo-Angiogenesis and Intraplaque Hemorrhages

Physiologically, the arterial media and intima are avascular; only the adventitia, the external layer of the wall, is vascularized (vasa vasorum). Thus, transport of soluble molecules and diapedesis of circulating cells are quite different between the arterial wall and the capillaries. In the capillaries, where pressure and velocity are low, oxygen and glucose transport is mainly due to diffusion, in which the concentration gradients are the driving force. In contrast, this transport is advective (convection) in the arterial wall, due to the hydraulic conductance driven by a pressure gradient that is more efficient than diffusion. Therefore, there is no phylogenic and developmental need for the presence of capillaries to ensure the energetic support of the wall media [1], and circulating cell diapedesis essentially occurs in the adventitia. Neo-angiogenesis within the wall is essentially associated with pathology. Due to the metabolism of retained phospholipids by phospholipase A_2_ (PLA_2_) in fatty streaks and fibroatheroma, arachidonic acid is generated and the cyclo-oxygenase pathway activated. This pathway produces prostanoids, including prostaglandin J2, which activate the expression of vascular endothelial growth factor (VEGF) by subjacent SMCs (growth factor gradient and outward convection) [58]. SMC-VEGF initiates neo-angiogenesis from the adventitia towards the plaque [59]. However, these neo-vessels remain frail because of the proteolytic micro-environment within the advanced plaques [60]. This neo-angiogenesis allows diapedesis of RBCs throughout the neo-endothelium toward the medial tissue. It is the main source of bleeding when neo-vessels reach the plaque core. These intraplaque hemorrhages also lead to clotting including fibrin formation and leukocyte retention, which increase both the proteolytic [61] and the oxidant activities of MPO by Fe^++^ [62] within the core, promoting plaque vulnerability [38]. These events are one of the most important causes of the evolution of atherothrombotic diseases towards clinical expression [63,64]. These intraplaque hemorrhages have been described in culprit plaques of different localizations: coronary arteries [29], carotid arteries [65] and infrarenal aorta (see below AAA).

### 5.4. In-Stent Neoatherosclerosis

Currently used drug-eluting stents (DESs), despite preventing SMC proliferation-induced restenosis, promote the development of in-stent neoatherosclerosis [66]. We recently observed that peri-strut micro-hemorrhages were the main pathological phenomenon associated the long-term implantation of DES [67], leading to the development of late in-stent vulnerable plaques and finally to delayed thrombosis [68,69]. These hemorrhages are mainly related to the mechanical mismatch between the elastic behavior of the arterial wall (pulsation) and the unalterable rigid property of the metal stent, acting at each systolo/diastolic motion (see below calcifications).

### 5.5. Consequences of Clot Integration

Besides bleeding, the integration of a clot within the arterial wall is a historic observational paradigm in human atherothrombotic disease. The famous Austrian pathologist C. Rokitansky (1804–1878) described the arterial atheromatous process as evolutive intimal blood deposits, including advective insudation of plasma proteins (convection) [70] and blood cell deposits and their integration within the intima [71]. In this context, the integration of RBC clotting is one of the main pathological phenomena [72] associating, as usual, RBC membrane release participating (not exclusively, as platelet membranes may also play a role [73]) in tissue enrichment in cholesterol and its crystallization, and associated oxidative processes due to hemoglobin and Fe^++^ release [47]. These early observations were reinforced by the study of E. Arbustini [36] showing that atherosclerotic fatty plaques can develop in pulmonary arteries only in cases of pulmonary artery hypertension (PAH) secondary to pulmonary embolism but not in cases of primary PAH. The observed pultaceous plaques consist of organizing thrombi, foam cells, cholesterol clefts, vascular angiogenesis, RBCs and sometimes calcifications, usually incorporated into the pulmonary arterial wall from the embolism.

Whatever the source, bleeding or thrombus integration, RBC retention within the wall and intratissue hemolysis participate actively in oxidation. High Density Lipoproteins (HDL) also transit through the wall, potentially more than LDL, but do not interfere with the early stages of human atherosclerosis. In advanced stages of atherothrombosis, the association of redox-active hemoglobin with neutrophil MPO is so powerfully oxidative that it oxidizes ApoA1 (the main apo of HDL) and separates it from its lipid cargo [74] in the arterial wall [75]. In this context, free oxidized ApoA1 (MW 30,000 Da) is filtrated by the glomerulus and metabolized in the proximal tubule via cubulin, decreasing the plasma bio-availability of ApoA1. This mural oxidative pathway potentially explains the observed decrease in HDL associated with the progression of oxidation, and atherothrombosis in human [76]. Conversely the observed decrease in tissue HDL is a biomarker of arterial wall oxidation due to the additional interaction of hemolysis with MPO release.

### 5.6. RBC Clotting in Aneurysms of the Abdominal Aorta

As described by S. Glagov in human autopsy studies [63], the infrarenal aorta is a privileged site for atherothrombotic disease. Arterial modeling during the fetal life in mammals is mainly adapted to frictional forces due to flow [1]. The heart and aorta are submitted to a high flow load during fetal life because they support both the vascularization of the fetal body (60%) and the placental circulation (40%). Therefore, the aortic diameter remains abnormally large after birth, with a low shear rate (16 s^−1^ in the aorta, ten times less as compared to 160 s^−1^ in common carotid artery) and a high tensional stress as the product of pressure multiplied by radius [1]. Therefore, the infrarenal aorta is highly sensitive to atherothrombosis including clotting, plaque hemorrhages and calcifications. However, due to the wide aortic diameter, these acquired pathologies can remain asymptomatic at this site, for long periods.

**Figure 4:**
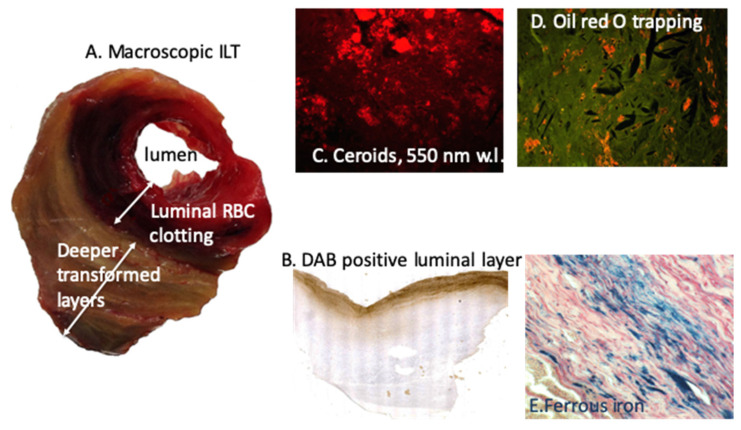
RBC-dependent oxidative stress in AAA: (**A**) macroscopic luminal view of the thrombus, with a predominant RBC fresh clotting at the interface with circulating blood; (**B**) diaminobenzidin (DAB) staining of RBC in the most luminal layer; (**C**) autofluorescent ceroids (550 nm), the wavelength of Hb; (**D**) oil red O staining of lipids (orange color); and (**E**) Perl’s blue staining of Fe^+++^, the storage redox inactive form of iron, bound to ferritin and transported by transferrin (recycling).

They may specifically and frequently evolve toward aneurysmal lesions (AAA), which are characterized by the development of luminal clotting (intraluminal thrombus, ILT), permanently renewed by the circulation [77]. RBC trapping and hemolysis are the most important components of the ILT in association with fibrin formation and neutrophil trapping [78]. We observed first the predominant role of RBC trapping by the ILT and the bipolar pro-oxidative role of this process, involving both the oxidation in the ILT, but also the outward transport of ferrous iron and associated oxidative reaction toward adventitia [79], able to initiate and promote adaptive immune response in the adventitia [80,81]. In these seminal studies, we observed the ability of luminal RBC entrapping to oxidatively precipitate DAB (Fe^++^), but also retention of cholesterol-rich membrane promoting cholesterol crystal cleft formation. In this context, iron is outwardly transported to adventitia and storage of Fe^+++^ (Perl’s) was observed at the interface with adventitia [79] (Figure 4). Thiobarbituric acid reactive substances (TBARS) markers of lipid oxidation, advanced oxidation protein product (AOPP) and 8-hydroxy-deoxyguanosine (8-OH-dG) products of DNA oxidation were intensively release by all AAA wall, including adventitia. Moreover, conditioned media of the most luminal layer of the ILT provoked a high level of ROS production and SMC apoptosis when incubated with cultured SMC, a process which was significantly rescued by Hb depletion or iron chelation (Deferoxamine). Moreover, the ILT has renewal and lytic dynamics, consumptive of platelets and fibrinogen, but also of circulating RBCs causing hemolysis and relative anemia [82]. This consumptive anemia is characterized by a decrease in RBC count and blood hemoglobin, associated with low circulating iron and transferrin, but high iron retention and hepcidin concentration, leading to altered iron recycling. Such consumptive anemia is predictive of poor patient outcome, and, in association with neutrophil MPO, of intensive oxidation dynamics [83]. As described above for advanced atherosclerosis, ApoA-1 is highly sensitive to oxidation and the ILT of AAA induces this oxidation [84] rendering HDL less protective for vascular tissue. Therefore, AAA evolution in humans is characterized by consumption of HDL by ILT oxidative dynamics, correlated with the decrease in plasma ApoA-1 and inversely correlated with AAA size and ILT volume [85]. HDL consumption by ILT oxidative dynamics is predictive of AAA growth, and HDL plasma levels were shown to be inversely associated with the need of surgical repair. Moreover, the HDL decrease observed in AAA patients is more intense than the decrease observed in patients with aorto-iliac atherosclerosis. These data are impressive, showing how much the constant renewal of the ILT with new RBCs and, potentially, neutrophils is redox-active, generating anemia with iron retention and further HDL degradation and ApoA-1 dysfunction. Therefore, the decrease in HDL associated with different localizations and forms of atherothrombosis provides evidence of heme-dependent oxidation during HDL transport through the wall [75]. In this context, magnetic resonance imaging (MRI) can detect iron as a black signal in the most luminal part of the ILT and at the wall/adventitia interface, both sites where ferric iron accumulates within phagocytes. This spontaneous signal can be enhanced by superparamagnetic status of ultra-small particle iron oxide (USPIO) [86,87].

### 5.7. RBCs and Vascular Calcifications

The relationship between valvular and arterial calcifications [88] and RBCs or Hb are bidirectional: calcifications promote hemorrhages and hemoglobin release promotes calcifications of elastic vascular tissues. The initiation of calcifications in soft vascular tissues is directly related to extracellular exposure of tissue-cell-derived anionic phosphates (PO_4_^3−^) on which the ionized soluble cationic calcium (Ca^++^) precipitates. This reaction leads to the formation of calcium-phosphate [Ca_3_(PO_4_)_2_], which polymerizes into solid hydroxyapatite crystals (mineralization). This passive process rapidly initiates a phenotypic switch of SMCs towards an osteoblastic phenotype [89]. In the context of soft viscoelastic tissues such as arterial and valvular walls, calcifications promote a mechanical mismatch between the high strain of the elastic tissue, measured by finite element analysis, and the solid crystals, inducing a distortion energy at the interface between solid calcifications and elastic tissues (von Mises stress) [90]. This distortion causes fatigue-like repeated microdamage such as microscopic tears or macroscopic hemorrhages at the interface between calcifications and elastic tissue where the distortion shear is maximal. When microcalcifications develop in the intima, they can cause some breaches, which may sensitize to plaque rupture [91,92]. If the calcifications develop deeper in the media, distortion forces can induce tears of the neo-vascularization (see above) and peri-calcification hemorrhages [67]. A similar scenario develops in the aortic valves (Figure 1(C2) and Figure 2B). Aortic valve diseases are initiated by transvalvular transport of plasma lipoproteins, mainly LDL and Lp(a), during diastole, due to the pressure gradient between the aorta and the intraventricular diastolic pressure. This is why the lesions always develop in the fibrosa of the aortic valve (ventricularis for the mitral valve). Similar to healthy arterial media, healthy valves are avascular tissues [93]. As in the early stages of atheroma, lipid accumulation in the fibrosa can promote the development of angiogenesis within the valves, a process always associated with aortic valve diseases. In this context, microcalcifications develop in the fibrosa and could cause neovascularization tears and hemorrhages, which accelerate the calcification process [94], because hemoglobin and its derivatives (heme, ferrous iron, free radicals and NF-kB activation) promote the release of exosomes and the osteoblastic differentiation of valvular interstitial cells [95] as well as of SMCs in the arterial wall [96]. Therefore, there is a vicious circle between calcifications and RBC/iron which promotes exponential development of both valvular and vascular calcifications in association with CV risk factors: aging, tobacco, dyslipidemia, etc. These data suggest that membrane-associated release of NO promotes an osteoblastic shift in SMCs [97]. In this study, the authors showed that, besides phospholipids, isolated RBC membranes are able to promote vascular calcifications, independently of hemoglobin and depending on NO release by membranes. Membranes from RBCs deficient in NO synthase have limited procalcifying effects. These data suggest that, besides phospholipid support associated of RBC membranes, NO release complementarily promotes an osteoblastic shift in SMCs [97]. SMCs phagocytosing RBCs release numerous microvesicles and exosomes exposing the amphiphilic pole of phospholipids [9].

### 5.8. Myocardial Infarction (MI)

Heme-iron homeostasis is paradoxical in cardiac diseases, depending on the environmental conditions: local myocardial iron retention and tissue toxicity in MI [98] and systemic anemia in heart failure (HF) [99]. The frequency of anemia is around 30% in HF patients and hemoglobin is inversely correlated to left ventricle ejection fraction. The pathophysiology of anemia is multifactorial and heterogenous [100], but associated with inadequate erythropoietin [101], a decline in circulating hepcidin and an iron deficit [100]. Low hepcidin independently relates to unfavorable outcome.

There are two sources of heme-iron within the heart: hemoglobin of circulating RBCs in the coronary circulation and myoglobin, a hemoprotein for oxygen storage within cardiomyocytes, which is released into the plasma during MI. MI is always variably associated with erythrodiapedesis and hemorrhagic transformation, leading to tissue iron retention. The importance of iron retention, detected by MRI, has a predictive value for adverse remodeling of the LV and evolution toward HF [102]. As reported above for arteries, this detrimental effect is directly related to the cytotoxicity of heme-iron and catalysis of free radical release. Beside the ischemic death of cardiomyocytes, ferroptosis (see above) is certainly an aggravating factor of cell loss, extending the dysfunctional area and promoting detrimental evolution toward congestive HF [103].

### 5.9. Stroke

The neurotoxicity of free hemoglobin is well known. Neurons do not survive more than 24 h of exposure to hemoglobin. Therefore, erythrodiapedesis and hemolysis in the brain tissue, hemorrhages and hemorrhagic transformation of ischemic stroke are highly aggravating. It has been recently demonstrated that secondary vasospasm (due to NO inhibition by Hb) and neurotoxicity (due to subarachnoid hemorrhage) were rescued by the infusion of Hp in the cerebral spinal fluid, by a direct inhibition and sequestration of Hb-Hp complexes (m.w. > 100 kD) [104]. With regard to intact RBCs, erythrophagocytosis by microglia [105] is usually insufficient to prevent free hemoglobin release and neurotoxicity. Similar to myoglobin in muscles, neuroglobin is a reserve form of hemoprotein able to store oxygen and protect the brain, at least in part, from hypoxic/ischemic insults [106]. Ferroptosis also plays a role in secondary cell death in hemorrhagic stroke [107,108] and selenium loading can partially prevent ferroptosis in experimental strokes [109].

## 6. Protection from RBC Injury: From Cells, Plasma and Antioxidant Molecules

### 6.1. Clearance of Senescent RBC Physiology/Pathology

Clearance by phagocytosis is the physiological mechanism by which spleen macrophages remove senescent (s)RBCs, allowing the renewal of circulating RBCs. Erythrocytes pass through the cords of Billroth and reticular cell-rich sinusoids in the red pulp (reticulo-endothelial system), which contains a large population of monocytes and macrophages (professional phagocytes). sRBC exposing PS are engulfed by these macrophages and metabolized, allowing iron transport by transferrin and recycling within bone marrow. However, this physiological system corresponds to a systemic homeostasis of blood RBCs. In contrast, tissue stromal cells, including arterial wall SMCs [110], valvular interstitial cells (VIC) in cardiac valves [95], interstitial fibroblasts in the myocardium, glial cells [105] in the brain and epithelial cells [111], are also capable of local tissue RBC efferocytosis. Many data exist showing that non-professional phagocytosis, involved in efferocytosis, is a general feature of cells in most tissues [112]. This point is important in pathophysiology, because it suggests that professional phagocytes, macrophages for instance, are not mandatory for tissue debridement by efferocytosis [113]. Primary cultures of arterial SMCs are able to clear (and metabolize) a load of 10^6^ senescent RBCs in five days, whereas fresh RBCs remained intact. Senescent RBCs were phagocytosed by SMCs [110], whereas fresh RBCs were not (Figure 3). This phagocytic capacity of SMCs confirmed previous results, showing that this SMC phagocytosis is dependent on PS exposure [114]. SMC phagocytosis of RBCs causes accumulation of lipids (from membranes) and ferrous iron, and it increases their production of ROS. RBC phagocytosis also induces expression of CD68 (phagolysosome) and the synthesis of HO-1 and ferritin. After RBC intracellular hemolysis, lipid and hemoglobin metabolisms completely diverge. Heme, having gotten rid of globin, is metabolized by HO-1 into biliverdine, CO and Fe^++^, which is oxidized into redox-inactive Fe^+++^. Ferric iron binds to ferritin and can be exported, stored or recycled under the control of hepcidin. Similar capacities of efferocytosis were observed with cultured VIC (Figure 3(B1))

### 6.2. Haptoglobin, CD 163, Hemopexin, Deferoxamine

Haptoglobin (Hp, liver synthesis and secretion) is the endogenous direct ligand-inhibitor of free Hb [115]. The complex can be measured in the plasma as a marker of hemolysis. Hp structure and functions is genetically determined. The Hp/Hb complex binds the scavenger receptor CD163 present on numerous cells, physiologically including the spleen reticulo-endothelial system, but also SMCs of the pulmonary artery [116]. To the best of our knowledge, the ability of the arterial wall, particularly SMCs, to clear Hp/Hb complexes has not yet been explored.

Hemopexin is the endogenous ligand-inhibitor of free heme [117]. The heme/hemopexin complex is endocytosed by low density related protein-1 (LRP-1), a poorly selective scavenger receptor [118] highly present in SMCs. LRP-1 is able to engulf numerous complexes, such as LDL, protease/antiprotease complexes, heme/hemopexin, etc. More than 40 possible ligands have been identified [2].

Deferoxamine is a pharmacological iron chelator able to enter the cell, bind free iron and remove it via excretion in urine and feces [119]. By this direct effect on iron, chelators are capable of limiting iron-dependent oxidative stress within tissues and cells [120].

### 6.3. Anti-Oxidants: Glutathione, Thioredoxin, Peroxiredoxin, SOD, Glutathione Peroxidases, Catalase, Paraoxonase

Redox imbalance in the arterial wall could be the result of high ROS production but also of decreased/altered antioxidant systems. The main antioxidants comprise catalase, paraoxonase (PON), superoxide dismutase (SOD), glutathione (GSH) and glutathione peroxidases (GPX) and proteins of the thioredoxin (TRX) family, including TRX, TRX-reductase and peroxiredoxins (PRDXs) [121]. The role of these proteins in chronic vascular diseases has been previously reviewed [122]. Hydrogen peroxide (H_2_O_2_) produced by autoxidation of Hb is a predominant ROS in RBCs [123]. While intracellular ROS are neutralized by the highly abundant cytosolic antioxidant systems in RBCs (catalase, GPX and PRDX), ROS associated with Hb oxidation (among them, heme degradation products) are mainly located on the membrane [123]. An increase in heme products has been found in the membrane fraction of sRBCs and of pathological RBCs (with less stable Hb) [124]. In this respect, we previously showed that both catalase and PRDX-2 are decreased in the membrane of RBCs from AAA patients [125]. Moreover, a decreased activity of membrane bound antioxidant systems has been suggested to potentially decrease RBC lifespan [126], which could lead to anemia. Moreover, a low level of GPX-1 activity in RBCs is associated with increased cardiovascular risk and with future cardiovascular events [127,128]. Moreover, other cells with high pro-oxidant content such as neutrophils also contain antioxidant enzymes, such as catalase and SOD. Interestingly, both enzymes were decreased in neutrophils from AAA patients [129].

Finally, antioxidants could also have a non-cellular source such as those present in plasma (vitamins) or associated with plasma components (e.g., lipoproteins). The role of vitamins in preventing CVD is still a matter of debate, but new data support the importance of the correct identification of a specific target population for this treatment, such as patients with diabetes mellitus (DM) and the Hp genotype 2-2 [130]. Interestingly, Hp-Hb deficient clearance in Hp 2-2 DM individuals results in increased Hp-Hb binding to ApoA1 on HDL, thereby tethering the pro-oxidative heme moiety to HDL [131]. In addition, the activity of PON-1, the main antioxidant enzyme associated with HDL, is decreased in both atherosclerosis and AAA patients [131,132]. Thus, all these data strongly support the hypothesis that redox imbalance in chronic vascular remodeling could be derived, at least in part, from reduced antioxidant activities of both cell and plasma sources.

## 7. Conclusions

Because of their abundance, blood RBCs maintain permanent physiological and/or pathological interference with CV tissues, mainly the arterial wall, cardiac valves, kidneys, myocardium and brain tissues. Physiologically, RBCs are the main determinant of blood viscosity and, therefore, of the frictional forces exerted by the circulating blood on the arterial wall. In pathology, acute and chronic local colliding of RBCs with the wall, causing tissue hemolysis, is a major source of redox-active iron. Fe^++^ is the major catalyzer of all the oxidative reactions in living cells and tissues. In this pathological context, the main future challenge is to explore how to protect SMC, the stromal cell of the arterial wall, against iron-dependent oxidative stress in conjunction with biomechanical stress.
